# A Function Accounting for Training Set Size and Marker Density to Model the Average Accuracy of Genomic Prediction

**DOI:** 10.1371/journal.pone.0081046

**Published:** 2013-12-05

**Authors:** Malena Erbe, Birgit Gredler, Franz Reinhold Seefried, Beat Bapst, Henner Simianer

**Affiliations:** 1 Animal Breeding and Genetics Group, Department of Animal Sciences, Georg-August University, Goettingen, Germany; 2 Qualitas AG, Zug, Switzerland; Auburn University, United States of America

## Abstract

Prediction of genomic breeding values is of major practical relevance in dairy cattle breeding. Deterministic equations have been suggested to predict the accuracy of genomic breeding values in a given design which are based on training set size, reliability of phenotypes, and the number of independent chromosome segments (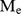). The aim of our study was to find a general deterministic equation for the average accuracy of genomic breeding values that also accounts for marker density and can be fitted empirically. Two data sets of 5′698 Holstein Friesian bulls genotyped with 50 K SNPs and 1′332 Brown Swiss bulls genotyped with 50 K SNPs and imputed to ∼600 K SNPs were available. Different k-fold (k = 2–10, 15, 20) cross-validation scenarios (50 replicates, random assignment) were performed using a genomic BLUP approach. A maximum likelihood approach was used to estimate the parameters of different prediction equations. The highest likelihood was obtained when using a modified form of the deterministic equation of Daetwyler *et al.* (2010), augmented by a weighting factor (w) based on the assumption that the maximum achievable accuracy is 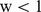. The proportion of genetic variance captured by the complete SNP sets (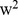) was 0.76 to 0.82 for Holstein Friesian and 0.72 to 0.75 for Brown Swiss. When modifying the number of SNPs, w was found to be proportional to the log of the marker density up to a limit which is population and trait specific and was found to be reached with ∼20′000 SNPs in the Brown Swiss population studied.

## Introduction

In dairy cattle, prediction of genomic breeding values (GBV) has become a basis for selecting young bulls which are not yet progeny tested. Often, conventional estimated breeding values, daughter yield deviations or deregressed proofs are used as quasi-phenotypes when training genomic prediction models ([1], [2]). The empirical correlation of predicted GBV and the (quasi-)phenotypes used that can be obtained via cross-validation or other empirical validation processes is often used as a measure for the accuracy of prediction (e.g. [2], [3], [4]). However, for selection purposes, we are more interested in the correlation of the predicted GBV and the true breeding value (TBV) which can be approximated from the obtained correlation of GBV and the quasi-phenotype ([5], [6]). In this study, we will refer to the correlation between predicted GBV and TBV (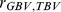) as the accuracy of genomic breeding value prediction.

For determining e.g. the required size of the training set or SNP density to achieve a predefined level of accuracy, it would be desirable to be able to assess the expected 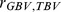 in advance for a GBV prediction study with a given design. Respective deterministic prediction equations have been suggested ([7], [8], [9], [10]). The approaches have been reported to fit well when applied to a limited number of data points in empirical studies ([10], [11], [12], [13]) and simulated data sets ([9], [10]). In these equations information on the number of animals in the training set, the heritability of the quasi-phenotype used, and the effective number of independently segregating chromosome segments (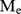) are the factors determining the accuracy. Daetwyler et al. [9] showed that the accuracy of the GBV obtained with genomic best linear unbiased prediction (GBLUP) models is independent from the number of underlying QTL. Therefore, this information is not accounted for in the deterministic equations when considering only results from GBLUP approaches. While all approaches referred to so far do not include information on the marker set used, Goddard et al. [10] suggested the number of markers as an additional parameter to account for in the prediction of accuracy.

Derivations of all these deterministic approaches imply that there are no relationship structures between the individuals. Wientjes et al. [13] studied the adaptability of such formulas to different simulation scenarios where selection candidates are related to the reference set in specific manner. They showed that the deterministic equation of [7] as well as the formula of [14] produced similar results for the reliability in comparison with reliabilities obtained with cross-validation also in scenarios where reference and validation individuals were highly related.

The number of independently segregating chromosome segments 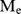 is a population parameter and is usually estimated based on assumptions of the effective population size (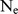) and the genetic length of the genome in Morgan (L). Different formulas ([8], [10], [15]) on how to determine 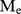 based on theoretical considerations lead to quite different 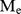, which has a major impact on the results of the deterministic prediction of the accuracy. Another possibility is to define the number of independent chromosome segments to be the reciprocal of the variance of the difference of the genomic relationship matrix and the numerator relationship matrix when complex family structures are in the data set ([10], [13]).

By using empirical accuracies obtained via cross-validation in a genomic prediction with real or simulated data, it is possible to determine 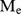 by rearranging the equation used for predicting of accuracy. With different levels of training set size this may lead to different estimates of 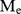 (see e.g. [9] with simulated data). Being a population parameter, 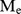 should have a constant value within one data set independently of the size of the training set used for its estimation, though. Daetwyler [16] proposed using a regression approach for overcoming this problem.

In our study, we suggest determining 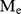 empirically based on a systematic multi-level cross-validation using a maximum likelihood approach and based on this, we will compare various deterministic prediction equations. We suggest a modified form of the deterministic prediction equation of [9] with the maximum accuracy that can be obtained with the given marker set as a further parameter, which will be shown to be a function of the natural logarithm of the marker density. All equations will be compared using two dairy cattle data sets of relevant size, and possible practical implications will be discussed.

## Materials and Methods

### Data Sets

To establish and test the methodology, we used a sample of 5′698 Holstein bulls, which were genotyped with the Illumina BovineSNP50 BeadChip. Single nucleotide polymorphisms (SNPs) with a minor allele frequency lower than 1%, with missing or non-autosomal position or a call rate lower than 95% were excluded. After filtering, there were 42′551 SNPs remaining for further analyses. Missing genotypes at these SNP positions were imputed using Beagle 3.2 ([17]). To study the influence of different marker densities, we reduced the number of markers to subsets of 30′000, 20′000, or 10′000, respectively. Markers in the subsets were chosen at random from the complete set.

All bulls used for this study had estimated breeding values based on progeny testing for somatic cell score and milk yield with an accuracy >0.84, which were used as quasi-phenotypes for the following analyses.

To test the proposed approach in a further data set and with different SNP marker density, we used a set of 1′332 Brown Swiss bulls which was partly genotyped with the Illumina BovineSNP50 BeadChip and partly with the Illumina BovineHD BeadChip with around 777 K. For the Brown Swiss bulls genotyped with the Illumina BovineSNP50 BeadChip, genotypes have been imputed to the Illumina BovineHD BeadChip based on a reference set of 727 Brown Swiss cows and 153 bulls using a combination of family and population imputation implemented in the software FImpute ([18]). After quality control, there were 627′306 SNPs available for further analyses. To study different marker densities, the set of markers was also decreased by using each 2^x^-th marker where x was 1, 2, …, 8.

Genotype and phenotype data is available from the authors on request.

### Cross-validation Strategy

Cross-validation was performed in different k-fold scenarios with k = 2, 3, …, 10, 15 or 20. This resulted in different sizes of training sets with different values of k. With a k-fold cross-validation, k-1 folds are used to predict the remaining fold and this procedure is repeated so that each fold is predicted once. Animals were assigned to the folds randomly. For the evaluation of the GBV prediction, the correlation 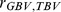 between predicted GBV and TBV was used, which was calculated as 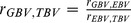 (e.g. [6]), where 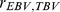 is the accuracy of the estimated breeding values, which we used as quasi-phenotypes. 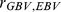 was calculated for each GBV prediction in a fold and then averaged over the k folds within a k-fold scenario. Each k-fold scenario was replicated 50 times, so that there were 50 values of 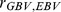 available for each k-fold scenario for further analyses.

### Genomic BLUP

Genomic breeding values were predicted using genomic best linear unbiased prediction (GBLUP) based on the model
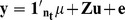
where 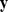 is a vector of quasi-phenotypes (in our case estimated breeding values of milk yield or somatic cell score, respectively) for all bulls in the training set, 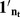 is a column vector of ones of length number of animals in the training set (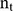), 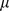 is the overall mean, 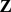 is the incidence matrix for the random genomic effect, 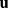 is a vector containing the random genomic effect (i.e. the genomic breeding value) for all animals and 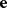 is a vector of random error terms. 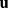 is assumed to be distributed 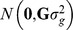 and 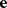 is assumed to follow 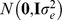. 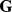 is the genomic relationship matrix following [14]. Since we wanted to study the effect of different number of markers, we built 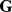 based on different SNP sets. For the basic scenario, we used all SNPs available after quality control (i.e. 42′551 SNPs for the Holstein Friesian data set and 627′306 for the Brown Swiss data set) while for the further scenarios 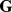 was based on a subset of the total available number of SNPs, namely on 30′000, 20′000 and 10′000 SNPs for the Holstein Friesian and 313′653, 156′827, 78′414, 39′207, 19′604, 9′802, 4′901 and 2′451 SNPs for the Brown Swiss data set, respectively. Variance components were estimated once with the respective complete data set in combination with a specific SNP set using ASReml 3.0 ([19]) and were then used for all respective runs using a subset of these data, but the same SNP set.

In the following, we will describe available deterministic equations for prediction of the level of accuracy from the literature and modifications of these formulas we will conduct.

### Equation of Daetwyler et al

Daetwyler et al. [9] presented an equation (D1) to predict the accuracy of a genome-wide genomic breeding value prediction:
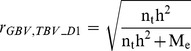
(D1)where 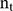 is the number of animals in the training set, 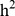 is the heritability of the observed trait and 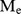 is the number of independently segregating chromosome segments. When estimated breeding values (EBV) from a conventional breeding value estimation scheme are used as quasi-phenotypes for genomic prediction, 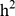 can be replaced by the reliability of the EBV. This is also true for all further prediction equations that will be described later. Daetwyler et al. [9] suggested using the definition of [8] to calculate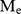, but we will take 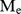 as a parameter not further determined in our study.

### Equation of Goddard et al

Goddard et al. [10] proposed a new equation for predicting the reliability of genomic prediction which also accounts for the number of markers used. The basic formula in this paper is
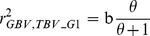
where
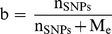
and
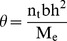



Goddard et al. [10] proposed a slightly different definition of 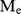 than [8] but we will not use any of them but keep 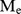 again as a population parameter to be determined empirically. Using those definitions, the prediction formula for the accuracy can be expressed as
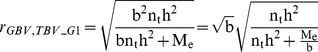
(G1)which is very similar to the one proposed by [9] but with the variable *b* included to account for the finite number of markers. Note that if 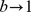, i.e. for a large number of SNPs and a limited number of 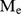, D1 and G1 become identical. Goddard et al. [10] suggested using also a correction factor due to a smaller error variance when using a multiple marker analysis rather than single marker analyses. They refer to [9] and present the optimal prediction equation (G2) for predicting the accuracy as
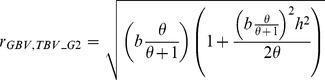
(G2)


### Modification of Daetwyler’s Equation

Assuming a finite 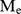 D1 will asymptotically approach 1 when 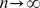. Daetwyler [16] stated in the general discussion of his PhD thesis that it may be useful to modify his prediction equation to deal with the fact that the marker density of the Illumina BovineSNP50 BeadChip might not be high enough to capture all genetic variation.

According to [20] the accuracy of the GBV as a predictor of the true breeding value component that is associated with the available marker set is a product of the square root of the proportion of genetic variance associated with the used marker set (*w*) and the accuracy of genomic breeding values assuming all causal variants are known and considered so that
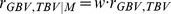



The factor 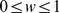 can be interpreted as the maximum accuracy that can be obtained with a specific SNP set when the size of the training set is infinite. Using this in model D1 leads to the modified equation (D2) of [9]

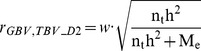
(D2)


### Modification of Goddard’s Equation

Equations G1 and G2 of [10] include also a weighting factor which accounts for the fact that not all genetic variance can be captured if the number of markers is limited. The authors of [10] defined this factor using the number of SNPs and the number of 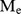 but this may not be the optimal factor. We thus wanted to study the results of prediction when using G2 in a modified form by setting 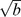 equal to our *w* and avoiding any further definition of *b*. This leads to prediction equation G3 defined as
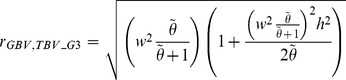
(G3)with
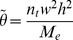



### Maximum Likelihood Approach

A maximum likelihood approach was used to determine the value of 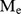 in equations D1, G1 and G2, or the combination of *w* and 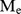 in equations D2 and G3 that provide the best fit of the respective model to our cross-validated data over all different training set sizes. We determined the most appropriate estimate of 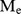 or *w* and 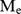, respectively, by maximizing the Likelihood function
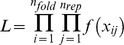
where 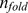 is the number of different k-fold scenarios, 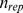 is the number of replicates within one scenario and 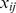 is the mean accuracy of prediction obtained by cross-validation in the 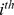 scenario in the 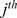 replicate. We assumed that 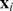 was approximately normal distributed with
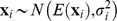
and observations were independent. 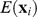 was derived from the respective model to predict the accuracy (i.e. D1*–*D2, G1*–*G3, respectively) and 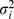 was assumed to be the empirical variance in the 50 observed values within the 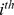 scenario. To ensure that the assumption of correlation coefficients being normally distributed random variables is not violated we tested all k-fold results with the 42′551 SNPs in the Holstein Friesian data set with a Shapiro-Wilk test [21].

Most of the parameters used in 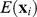 were determined by the empirical data, namely the heritability, number of animals in the training set and number of markers. Therefore, 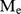 and *w* remain the only parameters in all considered equations to be adjusted. Searching for the maximal likelihood was done using the function “optimize” in R [22] for a one-dimensional search (i.e. for 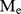 in equations D1, G1, and G2) and the function “optim” in R [22] for a two-dimensional search (i.e. for 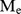 and *w* in D2 and G3).

### Predicting Prediction Accuracies

In many applications the prediction accuracy obtained with the data, especially the training set size, at hand is not sufficient. In such cases it would be desirable to be able to determine accurately the required training set size to achieve a pre-defined level of accuracy of genomic prediction. We tried to mimic this exercise to compare the usefulness of a model accounting for the fact that the finite marker set does not account for the full genetic variation (model D2) with that of a model not doing so (model D1). We used subsets of 4′000 Holstein-Friesian bulls to derive the optimal number of 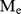 (in D1) or 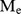 and *w* (in D2) and then predicted the accuracies for a training set in the size of the training set used for the 20-fold cross-validation runs with the whole Holstein Friesian data set (i.e. 5′413 bulls). For this we chose 4′000 bulls randomly out of the whole sample and performed a variance component estimation and all k-fold cross-validation runs (k = 2–10, 15, 20) for the different subsets. Afterwards, we maximized the likelihood as described above. Since there may be a sampling effect when using a random subset of 4′000 bulls, we repeated the whole procedure ten times so that we had predictions for ten different subsets of 4′000 bulls. The range of predicted values for a training set size of 5′413 bulls then was compared with the empirical accuracy from a 20-fold cross-validation with our whole data set, i.e. with a training set size of 5′413 bulls.

## Results

**Figure 1:**
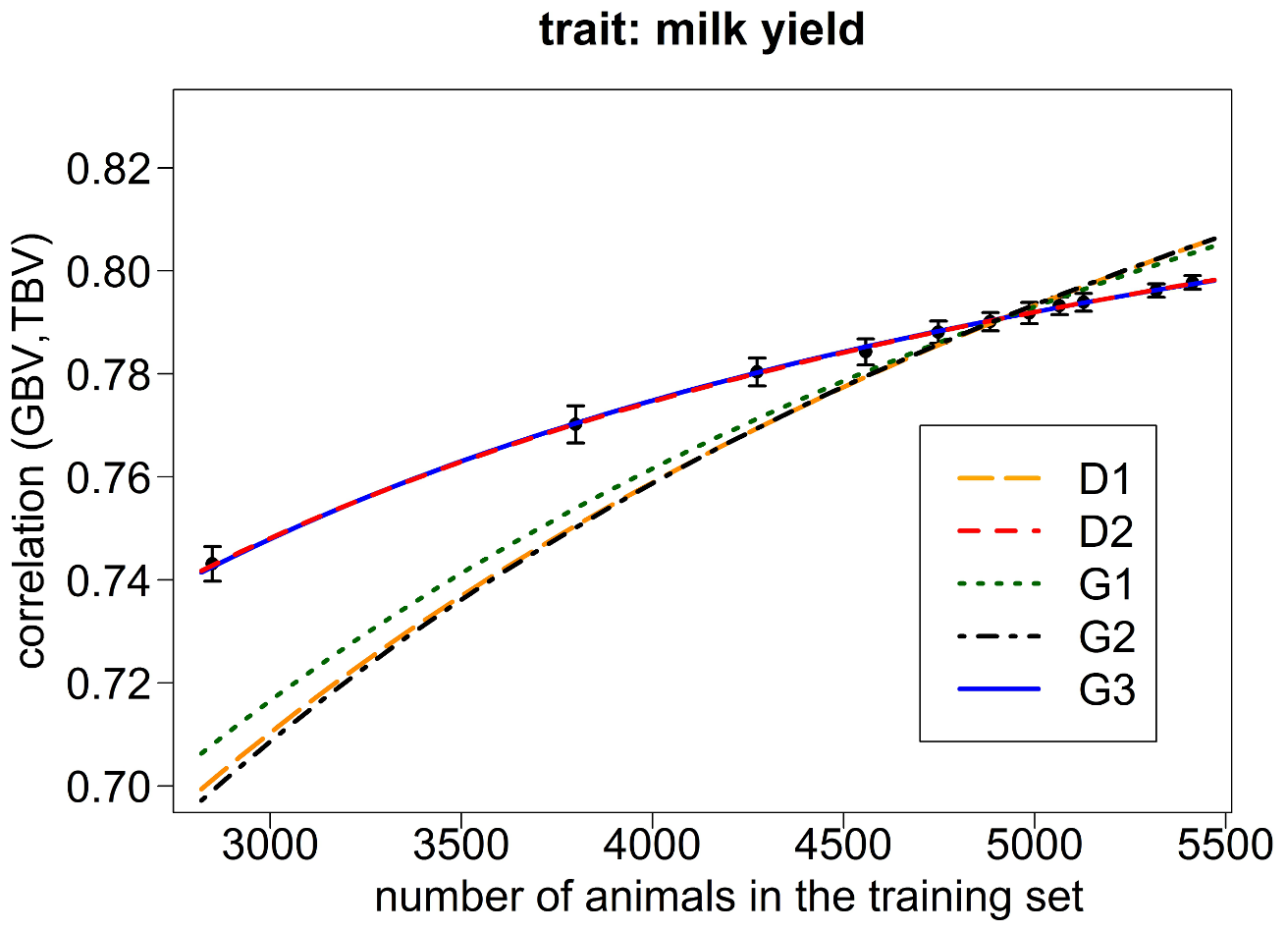
Empirical values and expected values of 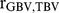 for milk yield in Holstein-Friesian data. Empirical values of 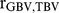 and expected values using the number of 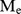 derived with a Maximum-Likelihood approach for the Holstein-Friesian data set in the original equation of Daetwyler et al. (2010) (D1) as well as in a modified form (D2) and in the equation of Goddard et al. (2011) without (G1) and with (G2) the proposed correction factor, respectively, and with the factor b not further determined (G3). For the empirical values, the mean and the standard deviation over the 50 replicates in each k-fold scenario of the Holstein-Friesian data set are shown.

**Figure 2:**
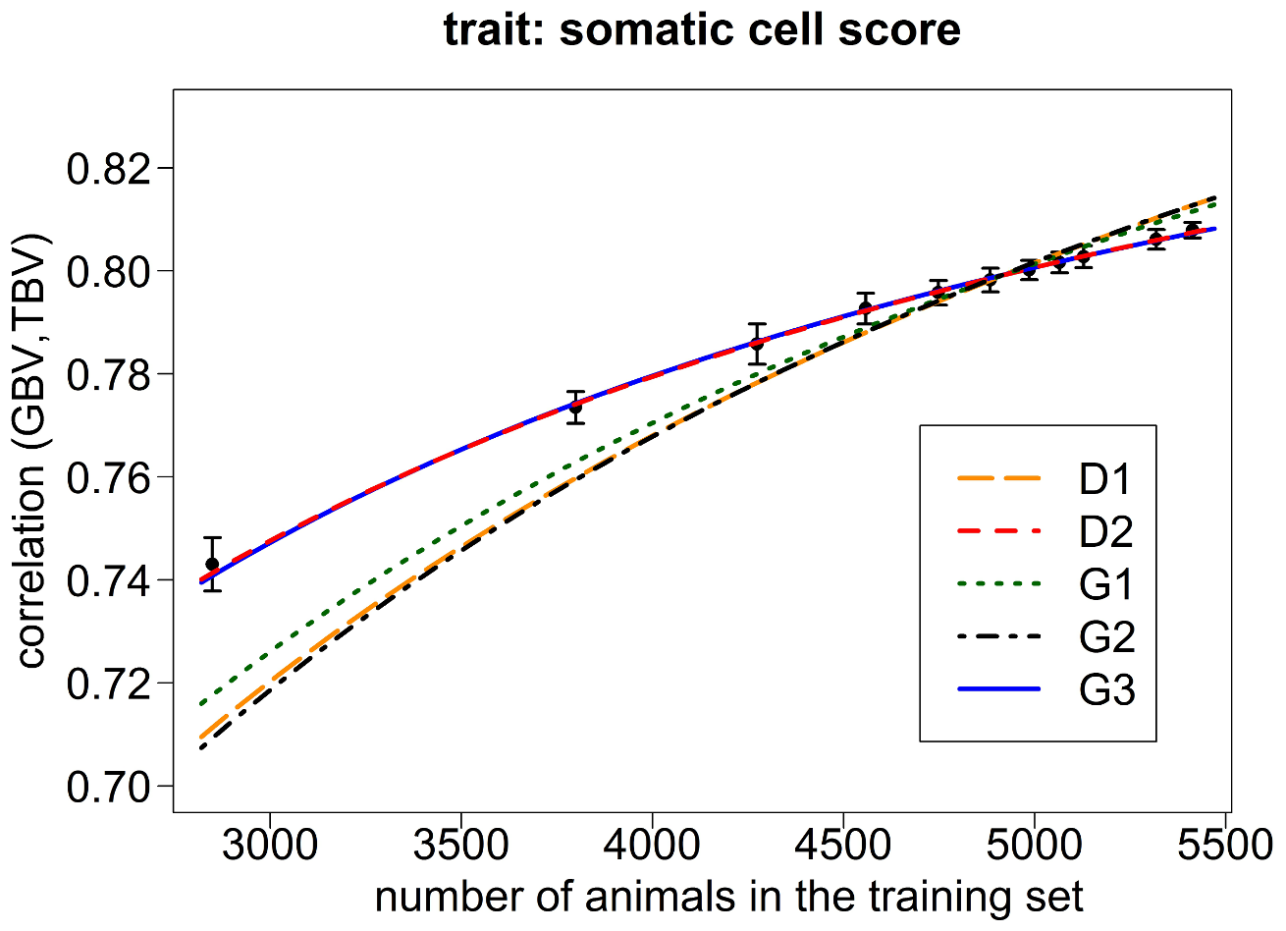
Empirical values and expected values of 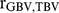 for somatic cell score in Holstein-Friesian data. Empirical values of 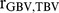 and expected values using the number of 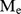 derived with a Maximum-Likelihood approach for the Holstein-Friesian data set in the original equation of Daetwyler et al. (2010) (D1) as well as in a modified form (D2) and in the equation of Goddard et al. (2011) without (G1) and with (G2) the proposed correction factor, respectively, and with the factor b not further determined (G3). For the empirical values, the mean and the standard deviation over the 50 replicates in each k-fold scenario of the Holstein-Friesian data set are shown.

The mean and standard errors of the empirical accuracies obtained from the different cross-validation schemes in the Holstein Friesian data are displayed in Figures 1 and 2 for the traits milk yield and somatic cell score. The mean accuracies (± standard errors) ranged from 0.743±0.0005 (0.73±0.0007) with a 2-fold cross-validation and training set size 2′849 to 0.798±0.0002 (0.808±0.0002) with a 20-fold cross-validation and training set size 5′413 for milk yield (somatic cell score).

Our observed accuracies were far away from the bounds of correlation coefficients (−1 and 1) and apparently normally distributed: The results of the Shapiro Wilk test showed that for all k-fold results with 42′551 SNPs in the Holstein Friesian data set the null hypothesis “normally distributed” was not rejected in a single case with p<0.01. Therefore, no further transformation of the data was necessary. Other approaches, like the least squares principle used by [12] to fit model D1 to sequence-based genomic predictions in *Drosophila melanogaster*, can also be used to estimate the model parameters and in our case would lead to very similar results (results not shown).

In the following, we will first describe the results for the estimates of 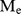 obtained based on the original equations from the literature [9], [10] and then based on the modified versions of these equations (i.e. with w added) with different numbers of markers.

Table 1 shows the numbers of 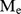 obtained by maximizing the likelihood of the empirical accuracies under equations D1, G1 and G2 for both traits. The estimates of 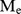 were of the same magnitude (∼between 2′000 and 2′800) with all methods while the likelihood obtained with G1 is highest for both traits. Not surprisingly, the estimates were similar for both traits since the empirical accuracies for milk yield and somatic cell score were very similar.

**Table 1:** Fitted values of the number of independent chromosome segments (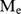) and weighting factors (w) with the Maximum-Likelihood approach and the corresponding natural logarithm of the likelihoods when using the Holstein-Friesian data set.

Method^1^	Trait	M_e_ fitted	w	% genetic variance captured	Ln(Likelihood)
D1	Milk yield	2783.2	–	–	−3912.5
D2	Milk yield	1045.6	0.875	76.6	2613.1
G1	Milk yield	2282.4	–	–	−1903.9
G2	Milk yield	2821.9	–	–	−4367.6
G3	Milk yield	904.9	0.869	75.5	2611.0
D1	Somatic cell score	2442.3	–	–	495.5
D2	Somatic cell score	1241.0	0.907	82.3	2512.9
G1	Somatic cell score	2036.2	–	–	1272.7
G2	Somatic cell score	2506.0	–	–	340.2
G3	Somatic cell score	1128.4	0.897	80.5	2508.7

1 D1 uses the formula of Daetwyler et al. (2010) to calculate the expected values of accuracy, G1 and G2 are based on Goddard et al. (2011) without and with the proposed correction factor, respectively. D2 is a modified equation of Daetwyler et al. (2010) while G3 is based on Goddard et al. (2011) with the weighting factor not defined like in the original publication but like in D2.

In Figure 1 and 2, the best curves of predicted accuracy under equations D1, G1 and G2 based on the respective maximum likelihood estimates of 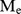 in the Holstein Friesian data set are shown for the traits milk yield and somatic cell score. None of these equations provided a curve of predicted accuracies that matched the empirical data to a sufficient extent. The results obtained under equations D1 and G2 are very similar while G1 provided a slightly better fit in accordance with the superior likelihood value for this model. Nevertheless, all equations led to a downward bias of predicted accuracies for small training set sizes while they showed an upward bias for large training set sizes.

Maximum likelihood estimates for *w* and 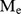 for the Holstein data set with the new equations D2 and G3 used for the calculation of the expectations of the accuracy are also presented in Table 1. The obtained likelihoods were dramatically higher compared to the conventional equations, with D2 slightly outperforming G3 with the present data sets. The estimates of 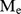 were clearly lower with both equations compared with the original equations and were in the range of ∼900 to ∼1′240 depending on method and trait. The optimal weighting factor *w* was in all cases between 0.87 and 0.91, suggesting that with the given marker set the accuracy of prediction will not approach 1 even if a very large training set is used. According to Dekkers (2007) the squared value of *w* represents the proportion of genetic variance associated with the markers which in our case would range between 75.5 per cent (milk yield with model G3) and 82.3 per cent (somatic cell score with model D2). This indicates that a large proportion, but not the complete genetic variation in our data set is captured by the SNP set at hand.

Figures 1 and 2 show prediction curves resulting from the optimal fit of the equations D2 and G3 for the traits milk yield (Fig. 1) and somatic cell score (Fig. 2) within the Holstein Friesian data set. For both traits and with both equations, the predicted accuracies fit the empirical data extremely well and in any case much better than with the conventional equations. By fitting two parameters (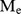 and *w*) the curves could accommodate a different slope of the empirical accuracy values more flexibly than with the one-parameter equations, which are bound to have their origin in 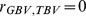 and asymptotically have to approach 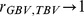.

Since we observed that only a specific fraction of the genetic variance was captured by the available SNP set we were interested in studying the effect of different SNP densities on the shape of the curve of expected accuracies and the respective parameters. Results of the maximum likelihood estimation using equations D2 and G3 with different marker set sizes in the Holstein Friesian data set are given in Table 2. We observed a decreasing trend in the weighting factor *w* when reducing the number of SNPs but the extent of the decrease was limited, so that even with 10′000 SNPs a high percentage of the genetic variance (71.2% for milk yield and 75.3% for somatic cell score, both with model D2) is captured and not much is gained by applying a more than four-fold SNP density. For the optimal number of 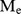 the trend was not that clear. It was also not expected that the number of 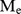 changes systematically in one direction since the same animals were used for all analyses. The likelihoods were in the same range for all reduced SNP sets compared to the full SNP set for both methods.

**Table 2:** Fitted values of the number of independent chromosome segments (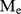) and weighting factors (w) with the Maximum-Likelihood approach and the corresponding natural logarithm of the likelihoods for different methods and different SNP sets when using the Holstein-Friesian data set.

Method^1^	Trait	No. of SNPs	M_e_ fitted	w	% genetic variance captured	Ln(Likelih.)
D2	Milk yield	10000	992.3	0.844	71.2	2576.4
D2	Milk yield	20000	1043.9	0.863	74.5	2600.0
D2	Milk yield	30000	1068.6	0.868	75.3	2594.4
D2	Milk yield	42551	1045.6	0.875	76.6	2613.1
G3	Milk yield	10000	791.6	0.838	70.2	2574.2
G3	Milk yield	20000	874.1	0.856	73.3	2597.2
G3	Milk yield	30000	904.1	0.861	74.1	2491.9
G3	Milk yield	42551	904.9	0.868	75.3	2611.0
D2	Somatic Cell Score	10000	1201.3	0.868	75.3	2457.8
D2	Somatic Cell Score	20000	1240.1	0.895	80.1	2496.0
D2	Somatic Cell Score	30000	1250.8	0.904	81.7	2512.3
D2	Somatic Cell Score	42551	1241.0	0.907	82.3	2512.9
G3	Somatic Cell Score	10000	993.5	0.861	74.1	2456.0
G3	Somatic Cell Score	20000	1093.3	0.885	78.3	2491.9
G3	Somatic Cell Score	30000	1127.0	0.894	80.0	2508.1
G3	Somatic Cell Score	42551	1128.4	0.897	80.4	2508.7

1 D2 is a modified equation of Daetwyler et al. (2010) while G3 is based on Goddard et al. (2011) with the weighting factor not defined like in the original publication but like in D2.

Based on our previous results, we next tried to describe the relationship between the estimates of w obtained and the underlying marker density.

**Figure 3:**
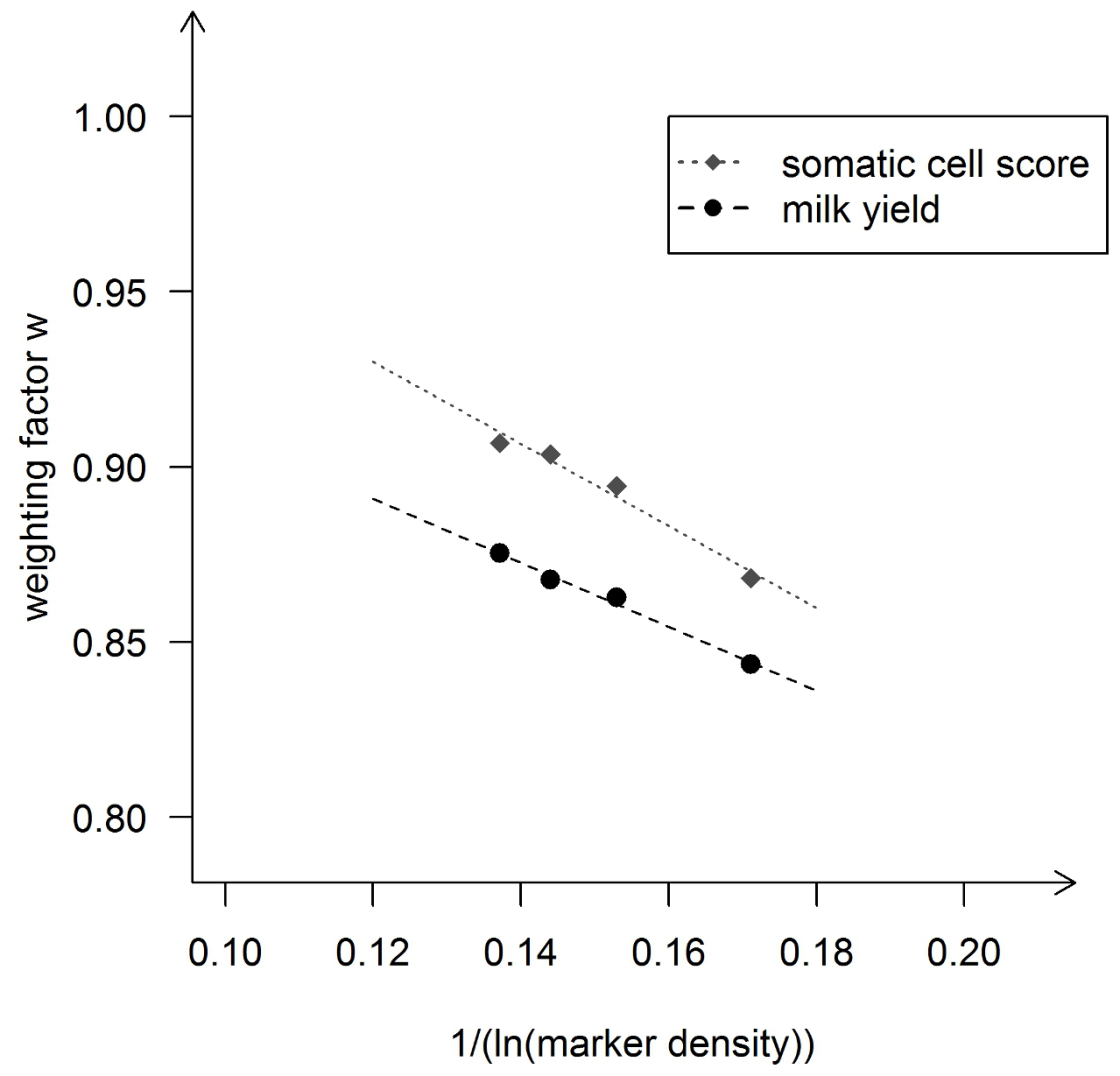
Regression of weighting factor w on the reciprocal of the logarithm of the marker density in Holstein-Friesian. Regression of the weighting factor w on the reciprocal of the natural logarithm of the marker density for the traits milk yield and somatic cell score in the Holstein-Friesian data set. The marker density was defined as the number of markers used divided by the length of the used parts of the genome in Morgan. The dots mark the values derived with the Maximum likelihood approach using the modified equation of Daetwyler et al. (2010) (D2) to describe the expected value of accuracy and the empirical data sets.

We hypothesize that the maximum accuracy that can be obtained, *w*, is a function of the natural logarithm of the SNP density. Using the Holstein Friesian data, we found that a function
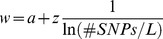
(1)where 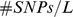 is the number of SNPs per Morgan, fitted our empirical data reasonably well (Figure 3). With an intercept of 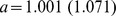 and a regression coefficient of 
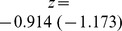
 for milk yield (somatic cell score), the coefficient of determination of the fitted regression line was 0.990 (0.971), and the regression coefficients were significant 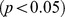 for both traits. Note that we had only four data points available, but nevertheless they showed a very clear trend. An intercept of approximately 1 could suggest that with an increasing SNP density (i.e. decreasing values of the reciprocal of the natural logarithm of the SNP density) the accuracy of genomic prediction asymptotically approaches 1. This result also suggested that it will be necessary to use multi-folds of a given marker density to obtain a substantial increase of the prediction accuracy.

**Figure 4:**
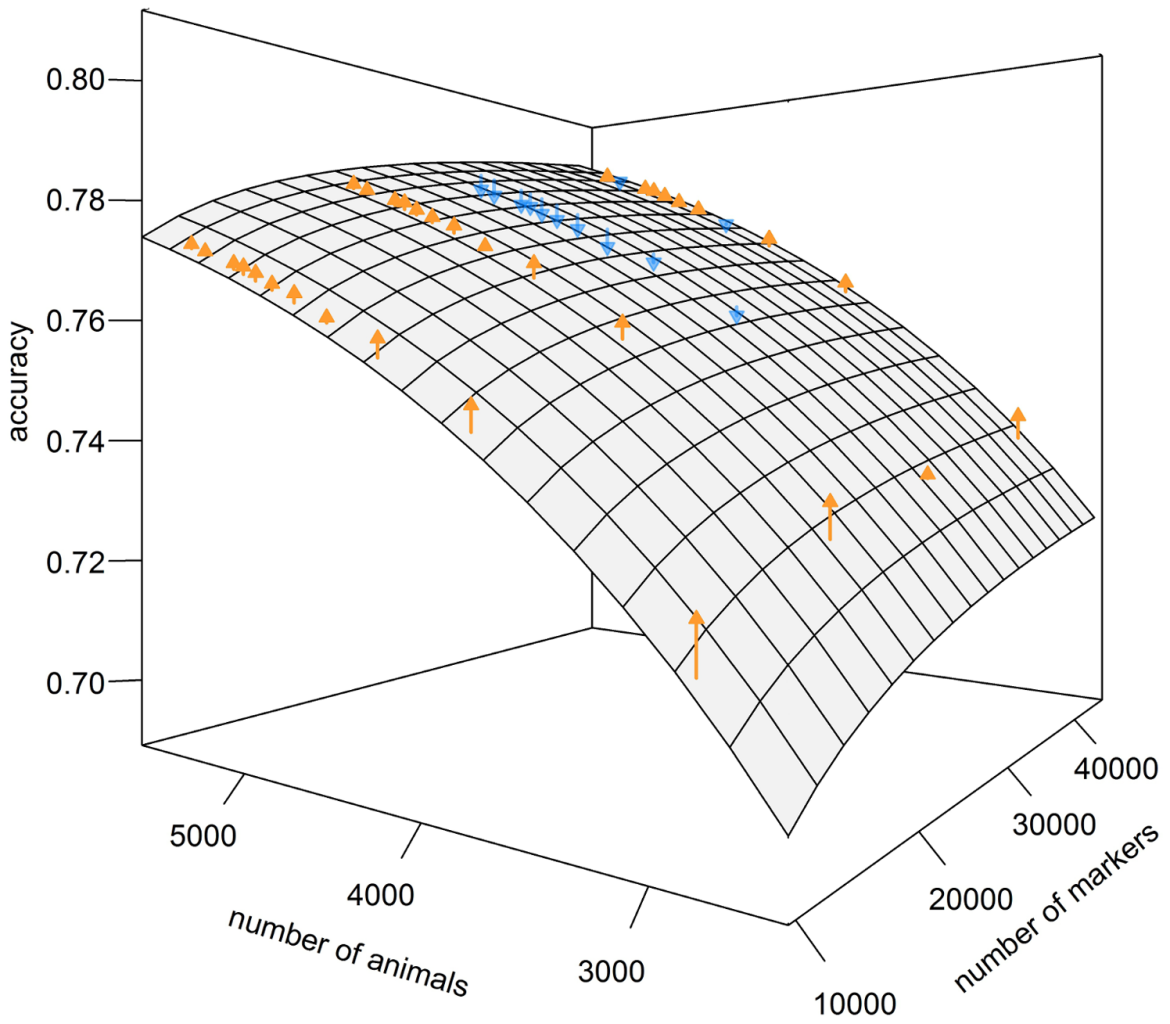
Predicted and empirical values of 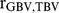 (grid) for different #SNPs and different #animals for Holstein-Friesian. Predicted values of 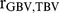 (grid) for different numbers of markers and different number of animals in the training set when using the modified equation of Daetwyler et al. (2010) (D2), an 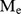 of 1′151.55 and a weighting factor of 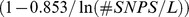. Empirical results obtained with cross-validation experiments with Holstein-Friesian data are symbolized by arrows. Orange arrows represent values that were higher than predicted while blue arrows indicate that empirical values were lower than the predicted ones.

As we had cross-validation results based on different marker densities available, we were also interested in finding a global function for estimating 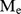 and a weighting factor including all available empirical results. Eq. [1] made it possible to find a global 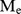 and a factor *z* depending on the marker density using our suggested maximum likelihood approach. We used D2 for the expected value with 
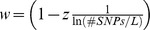
 and found the highest likelihood with 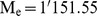 and 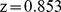. A comparison between predicted and empirical values is shown in Figure 4. It can clearly be seen that the empirical values deviate only slightly from the predicted values. Deviations are largest for small training set sizes and/or low marker densities.

**Figure 5:**
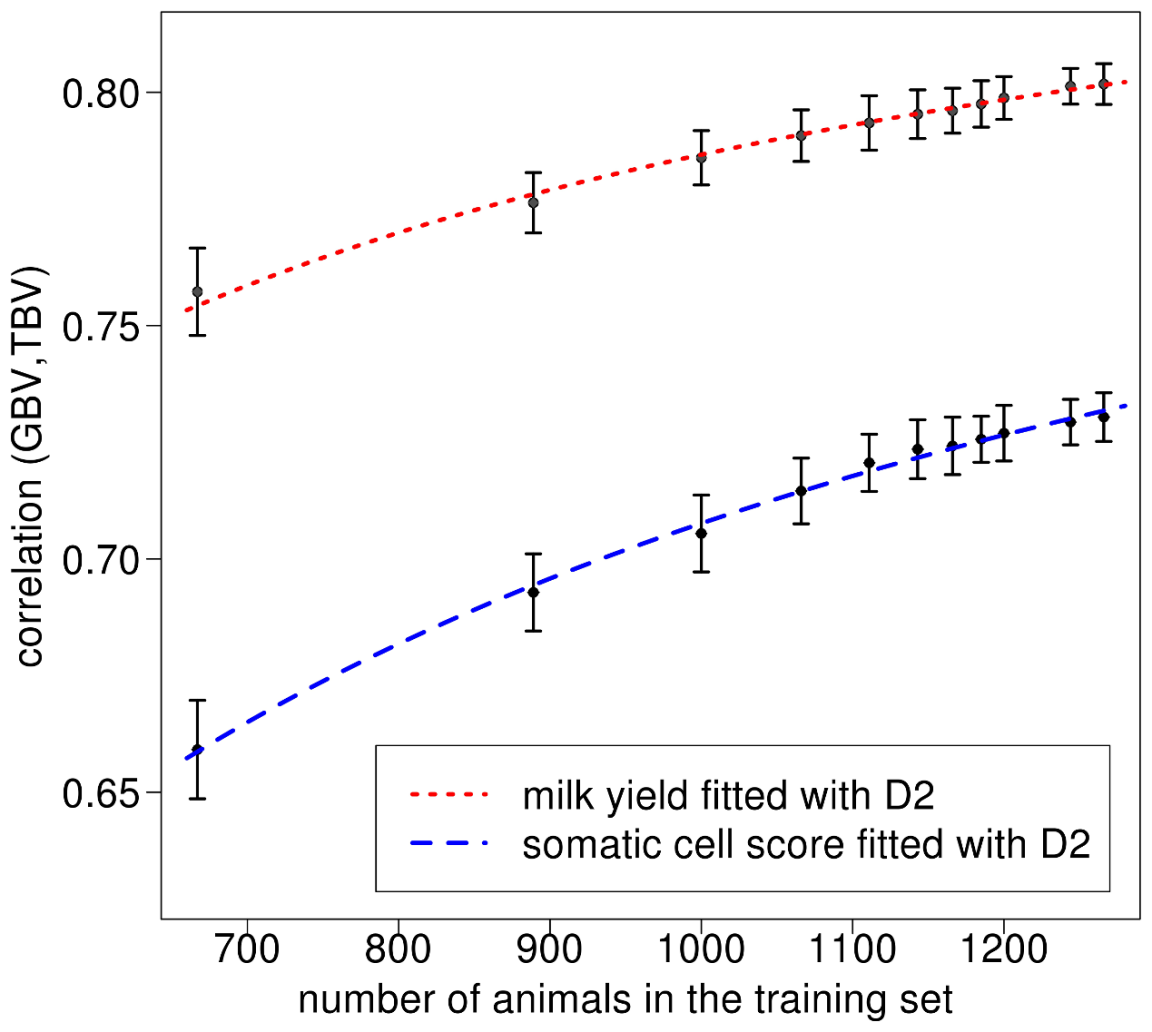
Empirical values and expected values of 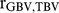 for milk yield and somatic cell score in Brown-Swiss. Empirical values of 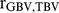 and expected values using the number of 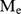 for the Brown Swiss data set derived with a Maximum-Likelihood approach in the modified equation of Daetwyler et al. (2010) (D2). For the empirical values of milk yield and somatic cell score in the Brown Swiss data set, the mean and the standard deviation over the 50 replicates in each k-fold scenario are shown.

To check the results in an independent data set, we applied the maximum likelihood approach based on D2 also on the Brown Swiss data set. Empirical values from the 2- to 20-fold cross-validation when using the full SNP set are shown in Figure 5. Mean accuracies (± standard errors) ranged from 0.757±0.0013 (0.659±0.0015) with a 2-fold cross-validation and training set size 667 to 0.802±0.0006 (0.730±0.0007) with a 20-fold cross-validation and training set size 1266 for milk yield (somatic cell score). Results of the estimation of the number of 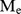 and *w* with different SNP sets can be seen in Table 3. Estimates for the number of 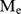 ranged from 148 to 214 for milk yield and from 277 to 419 for somatic cell score and were thus clearly lower in Brown Swiss than in Holstein Friesian. Estimates of the number of 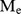 were smaller with milk yield than with somatic cell score as was also observed in the Holstein Friesian data set. The weighting factor *w* kept constant in both traits (∼0.87 for milk yield, ∼0.85 for somatic cell sore) when decreasing the number of markers up to a point of around 19′000 SNP from where on it decreased considerably. This indicates that the percentage of genetic variance captured with a given SNP set did not increase further when using more than 19′000 SNPs in this data set. Figure 5 shows the prediction curves with the optimized number of 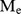 and an optimized *w* as well as D2 for modeling the expected accuracy for both traits and the full SNP set of 627′306 SNPs. As already seen with the Holstein Friesian data, D2 with optimized values for the number of 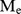 and *w* fitted the shape of the curve of empirical values very well.

**Table 3:** Fitted values of the number of independent chromosome segments (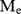) and weighting factors (w) with the Maximum-Likelihood approach and the corresponding natural logarithm of the likelihoods for method D2 and different SNP sets when using the Brown Swiss data set.

Trait	No. of SNPs	M_e_ fitted	w	% genetic variance captured	Ln(Likelih.)
Milk yield	2451	148.2	0.791	62.6	2111.2
Milk yield	4901	157.2	0.821	67.4	2108.2
Milk yield	9802	192.2	0.849	72.1	2078.3
Milk yield	19604	213.7	0.868	75.3	2075.8
Milk yield	39207	202.2	0.868	75.3	2085.4
Milk yield	78414	199.4	0.868	75.3	2090.9
Milk yield	156827	197.3	0.868	75.3	2095.2
Milk yield	313653	196.5	0.867	75.2	2094.0
Milk yield	627306	196.7	0.866	75.0	2092.2
Somatic Cell Score	2451	277.2	0.735	54.0	1904.7
Somatic Cell Score	4901	354.2	0.792	62.7	1910.0
Somatic Cell Score	9802	378.4	0.824	67.9	1971.9
Somatic Cell Score	19604	418.9	0.850	72.3	1979.7
Somatic Cell Score	39207	405.0	0.845	71.4	1978.6
Somatic Cell Score	78414	411.6	0.849	72.1	1983.0
Somatic Cell Score	156827	414.2	0.850	72.3	1981.4
Somatic Cell Score	313653	412.4	0.850	72.3	1982.0
Somatic Cell Score	627306	412.4	0.851	72.4	1983.9

**Figure 6:**
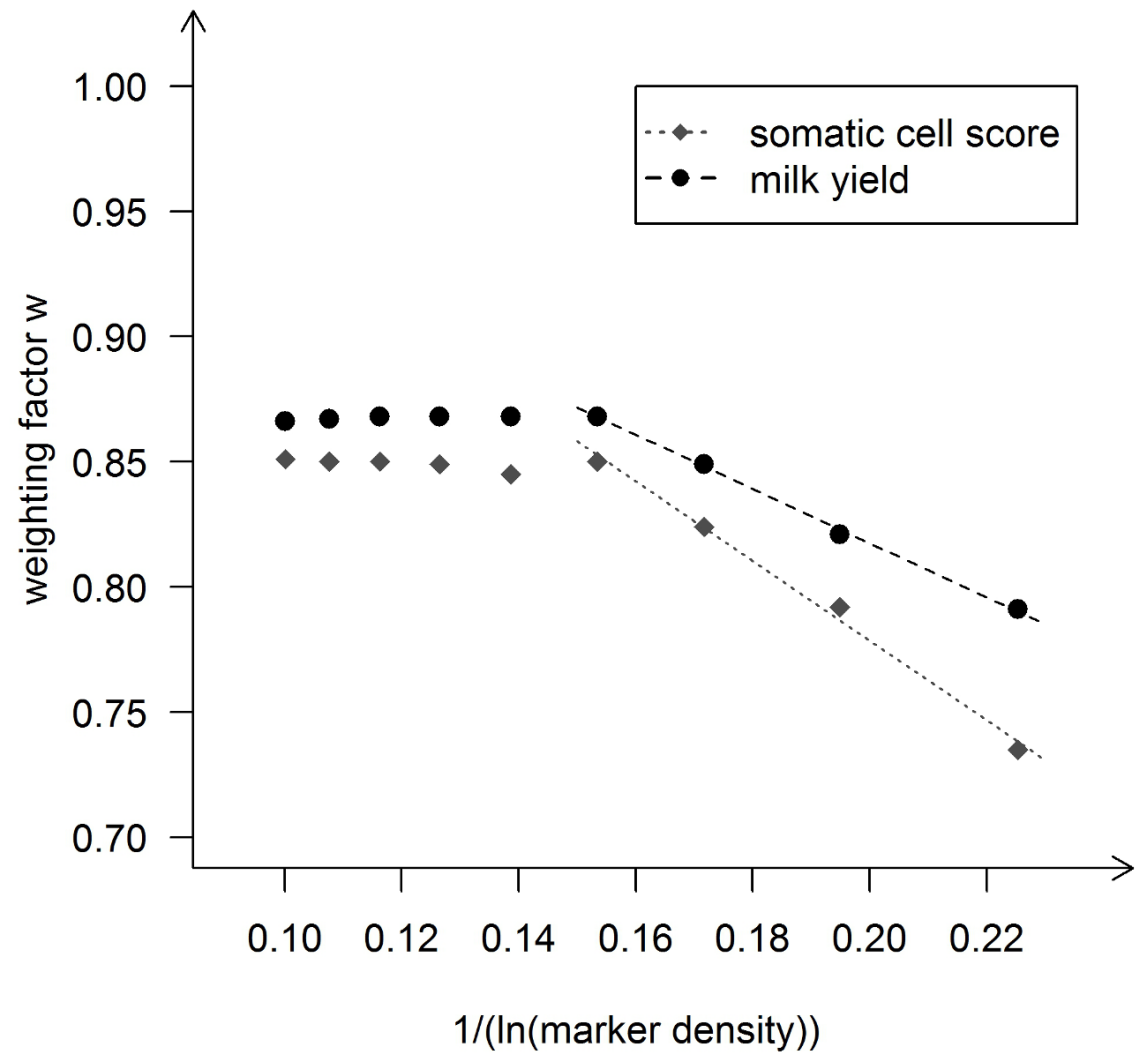
Regression of weighting factor w on the reciprocal of the logarithm of the marker density in Brown-Swiss. Regression of the weighting factor w on the reciprocal of the natural logarithm of the marker density for the traits milk yield and somatic cell score in the Brown Swiss data set. The marker density was defined as the number of markers used divided by length of the used parts of the genome in Morgan. The dots mark the values derived with the Maximum likelihood approach using the modified equation of Daetwyler et(2010) (D2) to describe the expected value of accuracy and the empirical data sets.

We also tested the relationship between the weighting factor *w* and the marker density for the Brown Swiss data set (same approach like in the Holstein Friesian data set). The results are shown in Figure 6. There seems to be a linear relationship up to a number of markers of around 20′000 SNPs (∼0.16 when expressed as 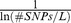). A linear regression model 
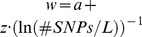
 with 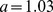 and 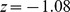 would lead to a coefficient of determination R^2^ of 0.998 for milk yield, for example. However, with any further increase of the marker density (i.e. smaller values on the x-axis), the weighting factor did not increase anymore but stayed on a constant level 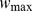 (e.g. 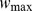
∼0.87 for milk yield). This pattern with a linear relationship first and constant values beyond a certain marker density was observed in both traits.

**Figure 7:**
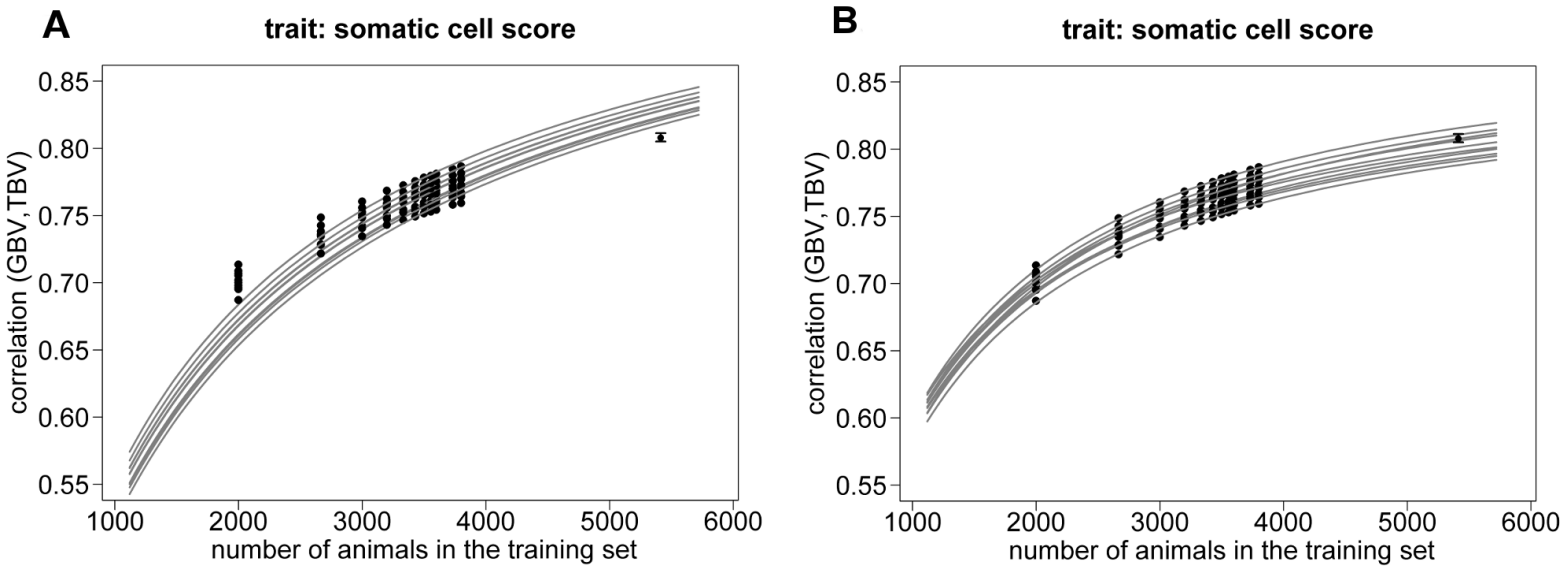
Predicted and empirical values of 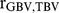 when extrapolating the accuracy. Empirical values of 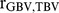 (black dots) of the ten replicates with different k-fold scenarios using 4′000 individuals and of the 20-fold runs of the fifty replicates using 5′698 Holstein-Friesian animals in total. Expected values (grey lines) use the number of 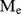 derived with a Maximum-Likelihood approach in the original equation of Daetwyler et al. (2010) (D1, Figure 7A) and in the modified equation of Daetwyler et al. (2010) (D2, Figure 7B).

Next we studied if our approach can be used to extrapolate the accuracy of prediction beyond the data set used to determine the model parameters. For this, the maximum likelihood approach was applied to ten data sets of 4′000 Holstein Friesian bulls which were the basis of cross-validation runs as described above. Figure 7A displays the resulting prediction curves obtained with model D1 for the trait somatic cell score, for milk yield the picture was very similar (results not shown). The curves varied over the data sets but were reasonably consistent in the level of accuracy and its slope over the different sizes of training sets. The fitted number of 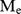 ranged between 2′000 and 2′356. When extrapolated to the training set size 5′413 (the one resulting from a 20-fold CV in the full data set), the expected prediction accuracy (averaged over the 10 replicates) was 0.828±0.007. The empirical accuracy obtained from the full data set (0.808±0.002) was clearly outside the range of predicted values obtained with the ten replicates. This suggests that model D1 (and similarly G1 and G2, results not shown) systematically overestimate the expected prediction accuracy when used for extrapolation beyond the training set size at hand.

In Figure 7B, D2 was used in the maximum likelihood approach to determine the optimal parameter for the prediction of accuracy based on the cross-validation runs with the ten different data sets of 4′000 bulls for the trait somatic cell score. The optimal weighting factor *w* ranged between 0.874 and 0.906 for the different data sets while the optimal number of 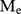 was between 979 and 1′195. These numbers were of the same magnitude as the optimal values we found when using cross-validation results with the total data set of 5′698 bulls. Using the proposed weighting factor made it possible to reflect the increase of accuracy when enlarging the number of animals in the training set. The empirical accuracies (0.808±0.002) obtained with a training set of 5′413 bulls was clearly within the range of accuracies (between 0.793 and 0.815) we would predict when using the parameters optimized for the ten data sets of 4′000 bulls and deviated only slightly from the average predicted value 0.803±0.007.

## Discussion

The aim of our study was to use empirical data to find a deterministic prediction equation for the accuracy of genomic breeding values that accounts for factors like sample size of the training set and marker density used that fits our data best. We used a maximum likelihood approach to validate different equations to predict the accuracy of GBV. We showed that the likelihood of our approach was best when the estimates of 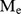 were obtained based on an expected value of the accuracy that also included a weighting factor reflecting the marker density used.

There are different possible reasons why the accuracy of genomic prediction with a specific SNP set may not reach one even if the number of training animals is infinite. First of all, only a fraction of the variance generated by QTL will be tagged by SNPs, i.e. the marker density is too low. Furthermore arrays like the Illumina BovineSNP50 BeadChip were designed such that the allele frequencies of the markers are more or less uniformly distributed ([23]) which leads to an underrepresentation of markers with very low minor allele frequencies. Since similar allele frequencies between marker and QTL are mandatory for obtaining high LD values and capturing the variance of the QTL, QTL with low minor allele frequency may not be represented adequately by the markers on a common SNP chip.

The weighting factor *w* can be interpreted as the maximum accuracy that can be achieved with the specific marker set in the population at hand assuming an infinite training set size. In our case, we found *w* to be in a range of ∼0.875 to 0.9 while the accuracies we could obtain with ∼5′700 bulls in our Holstein Friesian data set empirically were around 0.8. This means that most of the possibly achievable accuracy is already obtained when having ∼5′000 bulls in the training set. Genomic heritability (i.e. heritability in the GBLUP model) may be another good indicator of how much genetic variance is captured by the SNPs. Estimates of genomic heritability in our data sets (results not shown) were higher than the estimated squared w (representing the proportion of genetic variance captured by the SNPs), but behaved completely similar in trend (e.g. no increase in genomic heritability in Brown Swiss with additional markers from a number of ∼20′000 markers on) compared to w^2^.

Having the estimates of 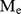 and w at hand, one could think about changes in accuracy when enlarging the training set size. Using model D2 with 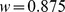 and 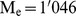 (values obtained for milk yield) we would need a training set size of 54′515 (10′246) animals to reach 99% (95%) of the possibly achievable accuracy with the given SNP density. Duplication of the number of animals in the training set from 5′000 to 10′000 would lead to a mean increase of accuracy of ∼0.04 from 0.79 to 0.83, while going from 10′000 to 20′000 animals would only lead to an increase of ∼0.02. Note that these considerations assume that a further random set of bulls (i.e. no specific groups like close relatives etc.) is used to enlarge the training set. In general, increasing the number of animals in the training set therefore will not add enough beyond a certain point when set in relation to the additional costs that incur for genotyping and phenotyping the required animals. Reliable knowledge about this case of diminishing returns is crucial when implementing or optimizing genomic selection programs.

Daetwyler [16] used a regression approach to estimate the maximum genetic variance captured by a SNP, which is the squared value of our weighting factor *w*. He observed four data points within US Holstein data sets for different training set sizes. However, he did not use different k-fold cross validation but validated his theory by augmenting the training set with new animals, including cows, to achieve larger training set sizes. The maximum genetic variance that is captured by the SNP set depends also on the population studied. Adding cows thus may bias the results since a higher genetic variance is expected in the cow population compared to the highly selected group of progeny tested bulls.

The maximum genetic variance found in his study was 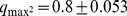 in US Holsteins for Net Merit with the 50 k SNP Chip which equates to a 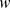 of ∼0.89. This is very close to our estimate in a European Holstein data set both for milk yield and somatic cell score. The weighting factor *w* in principle should be trait specific, but if conventional estimated breeding values (or equivalently de-regressed proofs or daughter yield deviations) are used as quasi-phenotypes for genomic prediction differences between traits should not be large as long as estimated breeding values are sufficiently accurate and homogeneous. Daetwyler [16] also suggested estimating 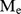 from model D1 based on results from real data [16] and simulated data [9]. For this, they rearranged D1 multiplied by the square root of 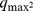 so that the number of 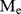 could be obtained directly. Their results for US Holsteins were in a range of about 900 to 1300 for the number of 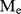 which is in the same range as the results we obtained with our Holstein data.

All numbers of 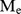 we derived in Holstein Friesian with D2 or G3 were similar or somewhat smaller than expected compared to the deterministic approach of [10] 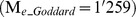 and clearly smaller than expected compared to the approach of [15] 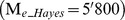 when assuming 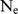 being 100 and the length of the autosomal genome being 29 Morgan. For Brown Swiss, the approach of [10] would clearly overestimate 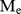 in comparison to what we found in the empirical data (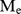 from 148 to 412). Hayes et al. [11] showed that expected accuracies were very close to empirical results from US Holstein Friesian cattle when using his definition of 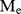 and an effective population size of 100, a length of the genome of 30 Morgan, and the original equation of [9]. For our data, however, the predicted accuracy using the assumptions of [15] would severely underestimate the accuracies observed in the cross-validation study (results not shown).

Goddard et al. [10] suggested the factor 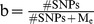 to estimate the proportion of genetic variance that can be explained by the markers, i.e. our factor *w* squared. With 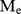 and 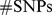 in the realistic range reflecting current applications in dairy cattle *b* will approach 1 very fast. For example, when 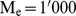 and 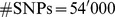, *b* would be 0.982 and therefore the square root of *b* (i.e.*w*) would be >0.99, which is clearly higher than found in experiments with real data ([2], [24], [25]) including this study.

We found a clear linear relationship between the reciprocal of the logarithm of the marker density and the maximal achievable accuracy (*w*) of the form 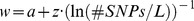 where 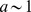 and *z* is a trait-specific regression coefficient. Such a linear relationship has also been found by [26] in simulated data. Since the relationship is linear to the log of the marker density, it is not surprising that the factor *w* which can represent the maximal achievable accuracy did not differ much between our runs with different number of SNPs in the Holstein data set. We could not study what will happen with further increasing the marker density in Holstein Friesian, since we did not have access to a sufficiently large set of individuals with high density marker genotypes.

Current results have shown that the accuracy of genomic breeding value prediction within breed did not increase significantly when using imputed 777 k SNP marker data rather than 50 k SNP data [24]. It seemed that also the proportion of genetic variance captured by the markers was only slightly higher. In our Brown Swiss data set, all bulls had 777 k SNP genotypes and we actually saw a stagnation of the percentage of genetic variance explained when the number of markers was greater than ∼20′000. This means that even with an infinite size of the training set the accuracy of prediction will not be better even if we use 30 times more markers. In Holstein Friesian, *w* still increased up to ∼40′000 markers roughly linearly with the logarithm of the marker density. It thus can only be assumed that the plateau has just not been reached for Holstein Friesian with the observed marker density, which remains to be verified once sufficiently large samples with high density genotypes are available for the Holstein Friesian breed.

The highest possible marker density is achieved when using whole genome sequence data in genomic prediction. In a data set of 157 inbred lines genotyped for ∼2.5 million SNPs, [12] found that the prediction equation D1 of [9] adapted for the special genetic model of *Drosophila melanogaster* was a good predictor for the accuracy of sequenced-based genomic breeding value estimation looking at different sizes of reference sets. Since the fit of the original equation of [9] to the empirical accuracies was excellent, it can be concluded that this massive SNP density (∼1 Mio SNPs/Morgan) recovers the complete genetic variability (i.e. 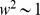) but in contrast to our study the small size of the reference set is the limiting factor in that case.

The results for the estimates of 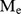 were very different in the two studied breeds. This was surprising because both are modern dairy breeds and rather similar results would have been expected. We thus assessed different characteristics of the two populations (Holstein Friesian and Brown Swiss) to identify potential causes for the difference in the pattern of observed accuracy functions. First, we calculated the effective population size 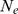 based on pedigree information and found values that were very similar for both breeds (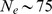, obtained with POPREP [27], based on [28]). Based on linkage disequilibrium (using markers available in both sets and formulas of [29] and [30]), estimates for 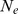 in 6 to 9 generations back was ∼133 in Holstein Friesian and ∼125 in Brown Swiss. Both analyses suggest that there is no difference between the two breeds regarding 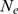. Furthermore, we studied properties of the genomic relationship matrix, namely the eigenvectors and eigenvalues of 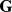 which reflect the degree of population substructure in the sample. To avoid a bias due to the number of SNPs used, we compared the genomic relationship matrix constructed with 42′551 SNPs for Holstein Friesian and 39′207 SNPs for Brown Swiss. The first and the second eigenvectors explained 14.36% (13.32%) and 6.29% (9.96%) of the variance in the Holstein Friesian (Brown Swiss) data set. The first 10 eigenvectors explained around 50% of the variance in both data sets. The differences between the structures of the eigenvectors in the covariance matrix therefore also seem to be negligible.

These results indicate that further parameters have to be found that can determine the proportion of genetic variance explained and the SNP density at which the plateau is reached. They also illustrate that calculating an expected value of 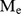 just based on the length of the genome and the effective population size may not be sufficient, since empirical values for 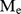 differ between traits within populations and even between populations with similar 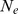 and the same length of genome. Furthermore, the results may also indicate that interpretability of population parameters (like e.g. M_e_) in such formulas can be limited when they are derived with the suggested goodness-of-fit-approach.

We further showed that model D2 allowed a realistic extrapolation of prediction accuracies with increasing training set sizes, while model D1 systematically overestimated the accuracy for a training set of 5′413 Holstein Friesian bulls when the model parameters 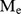 and *w* were derived with a subset of 4′000 bulls. The overestimation was not dramatic for this example, but 5′413 is not that much bigger than 4′000. However, if the difference between the number of individuals used for fitting the curve and the size of the reference set for which the accuracy is to be predicted increases, the upward bias will accumulate. Especially, as it is expected that number of animals in the training sets will increase in real studies up to ten thousands of training animals, it is critical that a prediction equation is able to fit the slope of the increasing accuracy correctly.

Equations to predict the accuracy of genomic breeding values are often derived for the simple case of a random set of animals that are not related (e.g. [8]) or show an ‘average’ relationship. In real cattle data, animals are often highly related and stem from specific selected groups, e.g. progeny-tested sires. A general equation, though, should be designed primarily as an indicator for a random animal out of a whole population (e.g. modern dairy cattle). Parameters like the number of 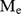 and *w* can be chosen in a way that they describe the underlying population and trait as good as possible, but it is not the goal to obtain exact predictions of accuracies for specific animals in the prediction set. As shown by many studies (e.g. [31]) the relationship between candidates and the training set, which also can be seen as a kind of population stratification, influences the accuracy in a non-random manner. Goddard et al. [10] showed how relationship structures can be used to estimate e.g. the parameter b but this works just in the case where animals have already been genotyped. Another idea on how to determine the maximum achievable accuracy has been recently proposed by de los Campos et al. (2013) [32]. They suggested an approach for the case of imperfect linkage disequilibrium between markers and QTL which is not depending on assumptions like unrelated individuals or parameters like M_e_. Further approaches still need to be developed for the “before data collection” case.

## Conclusion

We suggest a comprehensive model for the average accuracy of genomic breeding values and demonstrate how the model parameters can be estimated using a systematic cross-validation based on empirical data. Integrating all results, we suggest the model.
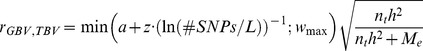
with the four parameters 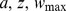 and 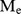, that can be empirically determined via systematic cross-validations as described in this study.

The suggested modification of the original equation of [9] led to a substantially improved fit of the predicted accuracies obtained with cross-validated data and showed its good prediction ability in the extrapolation to larger training sets. The maximum likelihood approach used for obtaining an estimate of the number of independent chromosome segments led to largely consistent values across different SNP sets. We also propose a function linking the maximally achievable accuracy of genomic prediction to the marker density, suggesting strongly diminishing returns when increasing the sizes of the SNP arrays, which confirms results obtained with different SNP densities in practical applications with dairy cattle.
